# Financial impact of reducing door-to-balloon time in ST-elevation myocardial infarction: a single hospital experience

**DOI:** 10.1186/1471-2261-9-32

**Published:** 2009-07-26

**Authors:** Umesh N Khot, Michele L Johnson-Wood, Jason B Geddes, Curtis Ramsey, Monica B Khot, Heather Taillon, Randall Todd, Saeed R Shaikh, William J Berg

**Affiliations:** 1 Indiana Heart Physicians, Indianapolis, Indiana, USA; 2 St. Francis Hospital and Health Centers, Beech Grove, Indiana, USA; 3 Curtis Ramsey and Associates, Indianapolis, Indiana, USA; 4 Emergency Physicians of Indianapolis, Beech Grove, Indiana, USA

## Abstract

**Background:**

The impact of reducing door-to-balloon time on hospital revenues, costs, and net income is unknown.

**Methods:**

We prospectively determined the impact on hospital finances of (1) emergency department physician activation of the catheterization lab and (2) immediate transfer of the patient to an immediately available catheterization lab by an in-house transfer team consisting of an emergency department nurse, a critical care unit nurse, and a chest pain unit nurse. We collected financial data for 52 consecutive ST-elevation myocardial infarction patients undergoing emergency percutaneous intervention from October 1, 2004–August 31, 2005 and compared this group to 80 consecutive ST-elevation myocardial infarction patients from September 1, 2005–June 26, 2006 after protocol implementation.

**Results:**

Per hospital admission, insurance payments (hospital revenue) decreased ($35,043 ± $36,670 vs. $25,329 ± $16,185, P = 0.039) along with total hospital costs ($28,082 ± $31,453 vs. $18,195 ± $9,242, P = 0.009). Hospital net income per admission was unchanged ($6962 vs. $7134, P = 0.95) as the drop in hospital revenue equaled the drop in costs. For every $1000 reduction in total hospital costs, insurance payments (hospital revenue) dropped $1077 for private payers and $1199 for Medicare/Medicaid. A decrease in hospital charges ($70,430 ± $74,033 vs. $53,514 ± $23,378, P = 0.059), diagnosis related group relative weight (3.7479 ± 2.6731 vs. 2.9729 ± 0.8545, P = 0.017) and outlier payments with hospital revenue>$100,000 (7.7% vs. 0%, P = 0.022) all contributed to decreasing ST-elevation myocardial infarction hospitalization revenue. One-year post-discharge financial follow-up revealed similar results: Insurance payments: $49,959 ± $53,741 vs. $35,937 ± $23,125, P = 0.044; Total hospital costs: $39,974 ± $37,434 vs. $26,778 ± $15,561, P = 0.007; Net Income: $9984 vs. $9159, P = 0.855.

**Conclusion:**

All of the financial benefits of reducing door-to-balloon time in ST-elevation myocardial infarction go to payers both during initial hospitalization and after one-year follow-up.

**Trial Registration:**

**ClinicalTrials.gov ID**: NCT00800163

## Background

The quality of health care by physicians and hospitals has received increasing interest due to well-documented deficiencies in the care delivered to patients [1]. Initially, this interest focused on public reporting of definitive outcomes such as mortality in patients undergoing coronary artery bypass grafting [2]. More recently, attention has shifted to markers of quality of care such as prescription rates for evidence-based drug therapies, documentation of education efforts for smoking and heart failure, and adherence to processes such as proper timing of antibiotic administration [3]. Interest in hospital quality reporting accelerated rapidly with implementation of the Hospital Quality Initiative by the Centers for Medicare and Medicaid Services (CMS). This program initially requested voluntary submission of 10 quality measures with the caveat that non-submission would result in a 0.4% decrease in Medicare payments. Participation in this program has been nearly universal with 99% of acute care hospitals submitting data to CMS, and the program has now evolved to include submission of 21 quality measures with future plans for additional measures [4].

It is widely assumed that improving the quality of care will decrease the cost of care. For example, the Leapfrog Group has asserted that adoption of care patterns found at high quality and efficient hospitals could save the healthcare system $5.6 billion dollars on the care of patients with acute myocardial infarction [5]. Similarly at the hospital level, some have advocated that there is a "business case" for quality, presuming that improved quality is associated with decreased hospital costs and improved hospital profitability [6-9]. However, these associations are not proof of a causal link between improved quality and decreased cost of care. In fact, at a broad level, there is a surprising paucity of research studying the causal relationship between improvements in the quality of care and the cost of care [10,11].

This increased focus on hospital quality reporting has particularly impacted cardiac care as measures involving myocardial infarction and congestive heart failure have received particular emphasis [3]. Of these specific measures, rapid door-to-balloon time in ST-elevation myocardial infarction (STEMI) is one of the most difficult to achieve, with most patients nationwide not achieving the recommended time of 90 minutes [12]. However, recent reports have highlighted specific strategies that can be implemented to reduce door-to-balloon time [13]. We recently showed that emergency department physician activation of the catheterization lab combined with a novel strategy of immediate transfer of the patient to an immediately available catheterization lab by in-house nursing staff reduces door-to-balloon time, leading to a reduction in myocardial infarct size and hospital length of stay [14]. In this report, we sought to determine whether this improvement in quality led to changes in the cost of care and whether the cost savings benefited payers or hospitals.

## Methods

### Study Design

Patient enrollment was conducted between October 1, 2004 and June 26, 2006 at St. Francis Hospital and Health Center (Beech Grove and Indianapolis, IN), a 591-bed tertiary care community hospital consisting of two campuses. We prospectively enrolled consecutive patients who presented to either Beech Grove or Indianapolis emergency department with STEMI who received percutaneous intervention within 24 hours of presentation [3]. On September 1, 2005 at 7:00 AM, we implemented a protocol mandating (1) emergency department physician activation of the catheterization lab and (2) immediate transfer of the patient to an immediately available catheterization lab by an in-house Emergency Heart Attack Response Team (EHART^®^), consisting of an emergency department nurse, a critical care unit nurse, and a chest pain unit nurse [14].

### Financial Analysis

Financial data from patients treated prior to the process change (Cardiology Activation/Routine Transfer period: October 1, 2004–August 30, 2005) were compared with financial data from patients treated after the process change (ED Activation/Immediate Transfer period: September 1, 2005–June 26, 2006). Revenue and cost data for all patients (including outliers) were analyzed. Hospital revenue indicates actual payments received from payers and is current as of September 2007 for initial hospitalization data and as of January 2008 for one-year financial data. Hospital cost data reflect the actual costs involved in the delivery of care to each patient and were determined by the hospital's cost-accounting software (Alliance for Decision Support, Avega. El Segundo, California). Both direct and indirect costs were determined. Contribution margin equaled revenue minus direct costs. Net income equaled revenue minus both direct and indirect costs.

The primary analysis included all patients with private insurance, Medicare, and Medicaid to focus on the financial data at the payer-hospital interface. Self-pay patients were excluded from the primary analysis, but were included in a secondary analysis (see Additional file 1). Financial data were stratified by payer status (private and Medicare/Medicaid) and were compared between the two time periods. Medicare and Medicaid were combined into one group as the number of Medicaid patients was small. Diagnosis related groups (DRG) were determined for all patients and standardized to reflect 2006 groupings. The DRG relative weight was determined for all patients. These were analyzed in unadjusted fashion and also analyzed after standardization to reflect fiscal 2006 values. Outlier payments were defined as hospital charges >$100,000 and hospital revenue >$100,000 and were compared between the two time periods. One-year follow-up was performed by combining financial data from the initial hospitalization with data from all hospital encounters (inpatient and outpatient) in the one-year post-hospital discharge. For patients with multiple hospitalizations for STEMI, the initial hospitalization was used as the index event and the subsequent hospitalization was included in the one-year financial follow-up data.

### Statistical Analysis

The statistical analysis plan was pre-specified prior to formal data analysis [14]. Time values are presented as medians with inter-quartile ranges and were analyzed using two-sample Wilcoxon rank-sum tests. Cost data are presented as mean ± standard deviation and were analyzed by two sample t-tests [15]. Categorical data are presented as proportions and were analyzed by Fisher's exact test. P < 0.05 was considered statistically significant. Stata Software was used for statistical analyses (version 8.2, College Station, Texas). Our institutional review board approved the study. The authors had full access to the data, take responsibility for its integrity, and have read and agree to the manuscript as written.

## Results

The two cohorts had well-matched demographics, initial presentation characteristics, and treatments as noted in the original paper [14]. Median door-to-balloon time decreased overall (113.5 minutes vs. 75.5 minutes, P < 0.0001), and treatment within 90 minutes increased from 28% to 71% (P < 0.0001). Mean infarct size decreased (Peak creatinine kinase: 2623 ± 3329 IU/L vs. 1517 ± 1556 IU/L, P = 0.0089), as did hospital length of stay (5 ± 7 days vs. 3 ± 2 days, P = 0.0097) [14].

The pattern of insurance coverage was similar (Table 1). In the primary analysis, hospital charges and hospital revenue both decreased with ED Activation/Immediate Transfer. Total hospital costs, direct hospital costs, and indirect hospital costs all decreased. A similar pattern was seen when the data was stratified by private insurance and Medicare/Medicaid status although the results were not statistically significant in the latter group due to small sample size (Table 1). The decrease in hospital revenue was greater than the decrease in hospital costs leading to a decrease in net income when stratified by payer status (private insurance and Medicare/Medicaid). For every $1000 reduction in total hospital costs, insurance payments (hospital revenue) dropped $1077 for private payers and $1199 for Medicare/Medicaid. However, there was slight numerical increase in overall hospital net income per admission due to a numerical increase in the proportion of private insurance patients.

**Table 1:** Health Insurance Status, Hospital Revenues, Costs, and Profit Margins

		Cardiology Activation Routine Transfer October 1, 2004–August 31, 2005 (N = 60)		ED Activation Immediate Transfer September 1, 2005–June 26, 2006 (N = 86)	
	N		N		P Value

**Health Insurance**	**60**		**86**		

Private		28 (46.7)		48 (55.8)	0.51
	
Medicare		21 (35)		29 (33.7)	
	
Medicaid		3 (5)		3 (3.5)	
	
Self Pay		8 (13.3)		6 (7)	

**Primary Analysis: All Patients, Except Self Pay**	**52**		**80**		

Hospital Charges		$70,430 ± $74,033		$53,514 ± $23,378	0.059

Hospital Revenue		$35,043 ± $36,670		$25,329 ± $16,185	0.039

Total Hospital Costs		$28,082 ± $31,453		$18,195 ± $9,242	0.009

Direct Costs		$20,533 ± $23,405		$12,862 ± $6,797	0.007

Cath Lab		$6,755 ± $3,727		$5,422 ± $2,026	0.009

Inpatient Nursing		$5,664 ± $9,302		$3,706 ± $2,783	0.079

Surgery		$3,269 ± $10,886		$401 ± $1,872	0.023

Pharmacy		$2,501 ± $2,805		$1,842 ± $1,972	0.115

Respiratory		$669 ± $1,819		$279 ± $965	0.111

Lab		$471 ± $783		$198 ± $165	0.003

Emergency Room		$421 ± $137		$514 ± $140	<0.001

Cardiology		$235 ± $396		$265 ± $160	0.553

Imaging		$205 ± $361		$92 ± $165	0.015

Supplies		$213 ± $337		$131 ± $210	0.088

Other		$128 ± $320		$12 ± $54	0.002

Indirect Costs		$7,549 ± $8,069		$5,333 ± $2,565	0.024

Contribution Margin		$14,511 ± $19,288		$12,467 ± $14,440	0.488

Net Income		$6,962 ± $16,818		$7,134 ± $14,451	0.95

**Private Insurance**	**28**		**48**		

Hospital Charges		$71,248 ± $50,121		$52,564 ± $26,917	0.038

Hospital Revenue		$46,500 ± $38,640		$33,005 ± $16,839	0.038

Total Hospital Costs		$29,994 ± $30,970		$17,462 ± $10,059	0.012

Direct Costs		$21,904 ± $22,709		$12,387 ± $7,406	0.009

Indirect Costs		$8,090 ± $8,274		$5,075 ± $2,769	0.023

Contribution Margin		$24,596 ± $20,316		$20,618 ± $12,668	0.296

Net Income		$16,505 ± $16,403		$15,543 ± $11,638	0.766

**Medicare/Medicaid**	**24**		**32**		

Hospital Charges		$69,475 ± $95,930		$54,939 ± $17,053	0.403

Hospital Revenue		$21,677 ± $29,711		$13,814 ± $3,095	0.142

Total Hospital Costs		$25,850 ± $32,529		$19,294 ± $7,884	0.276

Direct Costs		$18,933 ± $24,583		$13,575 ± $5,806	0.238

Indirect Costs		$6,917 ± $7,951		$5,719 ± $2,211	0.419

Contribution Margin		$2,745 ± $8,493		$239 ± $5,553	0.188

Net Income		$-4,173 ± $8,430		$-5,479 ± $7,286	0.537

Contribution Margin = Hospital Revenue-Direct Costs. Net Income = Hospital Revenue-Total Hospital Costs. All values per hospital admission.

**Figure 1:**
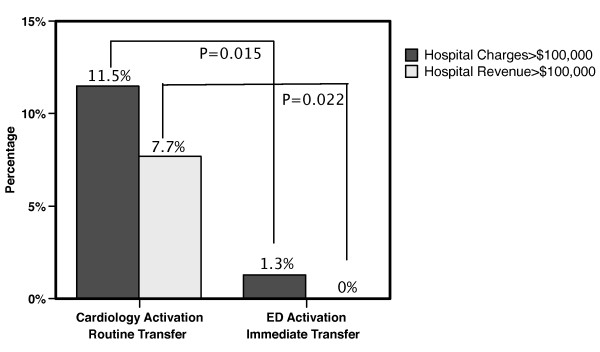
**Outlier payments defined as hospital charges>$100,000 and hospital revenue>$100,000 both decreased significantly with adoption of the ED Activation/Immediate Transfer process**.

The pattern of assigned DRG's for each admission was similar (Table 2). However, with implementation of the ED Activation/Immediate Transfer quality improvement, the DRG relative weight (which determines formal payment from Medicare) decreased significantly. The decrease was seen even when the data were standardized to reflect fiscal 2006 DRG relative weights. Similar patterns were seen when stratified by private insurance and Medicare/Medicaid. There was a reduction in outlier payments with the proportion of patients with hospital charges >$100,000 and hospital revenue >$100,000 both decreasing (Figure 1).

**Table 2:** Diagnosis Related Groups and Diagnosis Related Group Relative Weights

		Cardiology Activation Routine Transfer October 1, 2004–August 31, 2005 (N = 60)		ED Activation Immediate Transfer September 1, 2005–June 26, 2006 (N = 86)	
	N		N		P Value

**Assigned DRG** **(Adjusted 2006)**	**60**		**86**		

103		1 (1.7)		0 (0)	0.417
	
104		1 (1.7)		0 (0)	
	
106		5 (8.3)		3 (3.5)	
	
110		2 (3.3)		3 (3.5)	
	
555		12 (20)		20 (23.3)	
	
557		38 (63.3)		60 (69.8)	
	
558		1 (1.7)		0 (0)	

**DRG Relative Weights**					

**Primary Analysis: All Patients, Except Self Pay**	**52**		**80**		

Mean DRG Relative Weight (Unadjusted)		3.7479 ± 2.6731		2.9729 ± 0.8545	0.017

Mean DRG Relative Weight (2006 Weighting)		3.5979 ± 2.5718		2.9596 ± 0.8559	0.042

**Private Insurance**	**28**		**48**		

Mean DRG Relative Weight (Unadjusted)		3.7695 ± 1.7542		2.9573 ± 0.8793	0.009

Mean DRG Relative Weight (2006 Weighting)		3.6449 ± 1.7502		2.9443 ± 0.8817	0.023

**Medicare/Medicaid**	**24**		**32**		

Mean DRG Relative Weight (Unadjusted)		3.7226 ± 3.4972		2.9962 ± 0.8292	0.261

Mean DRG Relative Weight (2006 Weighting)		3.5430 ± 3.3263		2.9827 ± 0.8291	0.363

DRG = Diagnosis Related Group. DRG Relative Weight × Base Rate = Medicare payment. The drop in DRG relative weight with Medicare patients was 0.7264. Multiplied by St. Francis Hospital Base Rate of ~$5234 (average of $5171 for fiscal 2005 and $5297 for fiscal 2006) corresponds to reduction in payment of ~$3802 per admission for Medicare patients.

**Figure 2:**
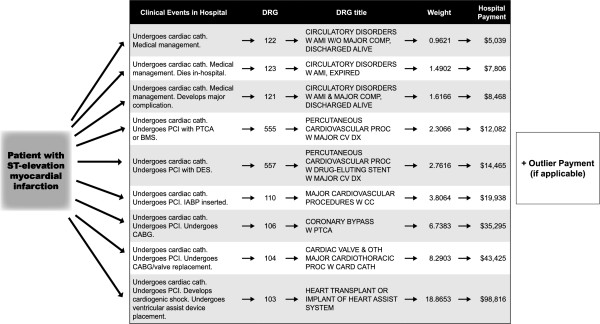
**Wide variation in payments from Medicare for ST-elevation myocardial infarction despite prospective payment system**. It is widely assumed that payments from Medicare are "fixed" due to prospective payment system. However, a patient presenting with ST-elevation myocardial infarction can have a wide variety of clinical events, which ultimately determine the assigned DRG, and can lead to a nearly 20-fold variation in payment from Medicare. Addition of payments from the outlier payment system can lead to even larger variations in hospital revenue. (Fiscal 2007 Medicare DRG Relative Weights and 2007 St. Francis Heart Center base rate of $5,238)

Patients were assigned “surgery” DRG’s not only for CABG, but also for ventricular assist device placement (DRG 103), mitral valve replacement (DRG 104), and intra-aortic balloon pump placement (DRG 110) (Figure 2 and Table 3). The proportion of patients undergoing CABG for complete revascularization in the absence of cardiogenic shock was similar (7.7% vs. 2.5%, P = 0.166). The exclusion of these patients undergoing CABG for complete revascularization in the absence of cardiogenic shock did not impact our conclusions as there remained significant decreases in insurance payments, total hospital costs, and diagnosis related group relative weight (Insurance payments: $34,499 ± $37,919 vs. $24,888 ± $15,265, P = 0.048; Total hospital costs: $27,060 ± $32,391 vs. $17,789 ± $8975, P = 0.018; Net Income: $7440 vs. $7099, P = 0.902; Diagnosis related group relative weight: 3.4514 ± 2.5663 vs. 2.8687 ± 0.5564, P = 0.055). These results were consistent with the primary analysis indicating that a numerical difference in patients requiring CABG for complete revascularization in the absence of cardiogenic shock could not explain our results.

**Table 3:** Characteristics of Patients with "Surgery" Diagnosis Related Groups

ED Activation/Immediate Transfer	IRA	Cardiogenic Shock	Left Main	3-V-CAD	Primary Surgery	DRG Weight	Total Charges
NO	RCA	Yes	Yes	No	CABG, VAD	19.5514	$516,563

NO	LAD	Yes	No	No	MVR, Aneurysm Resection	7.9180	$282,179

NO	LAD	No	Yes	Yes	CABG	7.3062	$64,707

NO	RCA	No	No	Yes	CABG	7.3062	$100,666

NO	RCA	Yes	No	Yes	CABG	7.3062	$149,503

NO	RCA	No	No	Yes	CABG	7.3062	$76,475

NO	RCA	No	Yes	Yes	CABG	7.3062	$79,628

NO	LAD	Yes	No	No	IABP	3.9587	$64,640

NO	LAD	Yes	No	No	IABP	3.9587	$125,504

YES	LAD	No	No	Yes	CABG	7.0346	$83,786

YES	LCx	Yes	No	No	CABG	7.0346	$208,978

YES	LAD	No	Yes	No	CABG	7.0346	$97,339

YES	LAD	Yes	No	No	IABP	3.9587	$51,238

YES	RCA	Yes	Yes	Yes	IABP	3.8417	$69,931

YES	LAD	Yes	No	No	IABP	3.8417	$91,175

Review of the "surgery" DRG's and of the highest resource utilization cases reveal that surgery (including non-CABG procedures) was commonly used in response to the development of post-myocardial infarction cardiogenic shock and not simply for coronary artery bypass grafting to allow for complete revascularization.

IRA = Infarct Related Artery, 3-V-CAD = Three Vessel Coronary Artery, DRG = Diagnosis Related Group, LAD = Left Anterior Descending Artery, LCX = Left Circumflex, Rca = Right Coronary Artery, CABG = Coronary Artery Bypass Grafting, VAD = Ventricular Assist Device, MVR = Mitral Valve Replacement, IABP = Intra-aortic Balloon Pump

The Medicare base rate to our hospital increased by 2.4% ($5171 in fiscal 2005 to $5297 in fiscal 2006) while private payer contracted payment rates increased in the range of 6–8% per year over the course of our study. Thus, our large decrease in actual hospital revenue per patient (Table 1 – a 36% decrease from Medicare/Medicaid and a 29% decrease for Private Payers) occurred even though there was a modest increase in the payment rates from government and private payers. Thus, the decreasing intensity of care associated with improved door-to-balloon time (i.e. reduced DRG Relative Weight, reduced charges, reduced outliers) was the primary determinant of reduced insurance payments.

**Figure 3:**
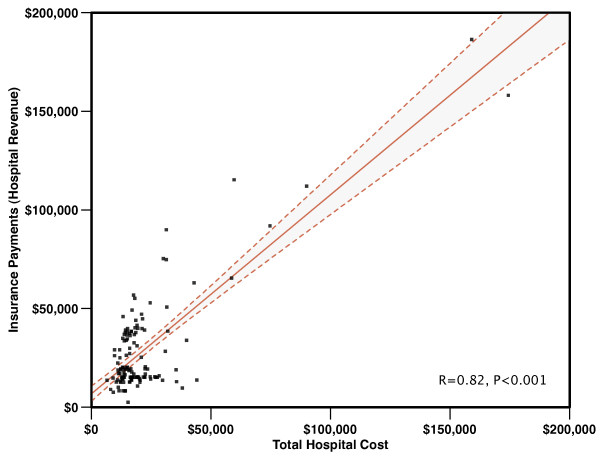
**Relationship between total hospital costs and insurance payments (hospital revenue) for study cohort**. There was a linear relationship between total hospital costs and insurance payments (hospital revenue); for every $1,000 reduction in total hospital costs, there was $1,009 reduction in insurance payments (hospital revenue). Thus, even with a significant proportion of patients treated by prospective payment (Medicare/Medicaid), there remains a strong relationship between costs and revenue. Dashed lines are 95% confidence intervals.

**Figure 4:**
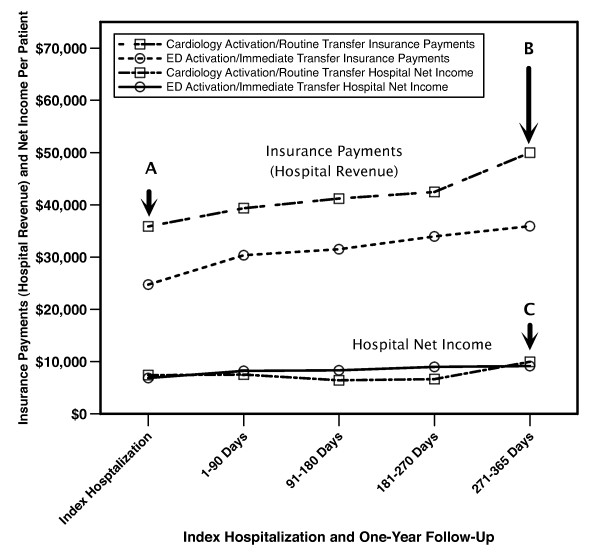
**Time course of financial outcomes from payer and hospital perspective**. (A) After the index hospitalization, there was a more than $10,000 reduction in insurance payments (hospital revenue) per patient. (B) After one year of follow-up, the reduction in insurance payments (hospital revenue) increased to greater than $14,000 per patient. (C) Despite marked improvement in hospital cost structure (Tables 1 and 3), hospital net income showed a slight numerical decrease as the decrease in revenue exceeded the cost reductions. Post-discharge one-year financial data include both outpatient and inpatient hospital encounters.

The relationship between total hospital costs and insurance payments is shown in Figure 3. For every $1000 reduction in costs, there was a $1009 reduction in insurance payments. In the one-year follow-up, two patients (one Private, one Medicare/Medicaid) from the Cardiology Activation/Routine Transfer period and three patients (two Private, one Self-Pay) from ED Activation/Immediate Transfer period had multiple STEMI hospitalizations. Thus, a total of 50 patients were included in the one year Cardiology Activation/Routine Transfer financial analysis and 78 patients were included in the one year ED Activation/Immediate Transfer financial analysis. One-year follow-up showed continuation in the patterns seen in the initial hospitalization analysis with incremental reductions in insurance payments (hospital revenue) ultimately exceeding $14,000 per patient with a slight numerical decrease in hospital net income (Table 4 and Figure 4).

**Table 4:** One-Year Financial Outcomes: Combination of Initial Hospitalization with All Hospital Encounters One Year Post-Hospital Discharge

		Cardiology Activation Routine Transfer October 1, 2004–August 31, 2005 (N = 60)		ED Activation Immediate Transfer September 1, 2005–June 26, 2006 (N = 86)	
	N		N		P Value

**Primary Analysis: All Patients, Except Self Pay**	**50**		**78**		

Hospital Charges		$101,862 ± $92,919		$77,215 ± $41,706	0.043

Hospital Revenue		$49,959 ± $53,741		$35,937 ± $23,125	0.044

Total Hospital Costs		$39,974 ± $37,434		$26,778 ± $15,561	0.007

Direct Costs		$29,031 ± $27,568		$18,967 ± $11,831	0.005

Indirect Costs		$10,943 ± $9,909		$7,811 ± $4,021	0.014

Contribution Margin		$20,928 ± $35,469		$16,970 ± $19,149	0.415

Net Income		$9,984 ± $32,175		$9,159 ± $18,847	0.855

**Private**	**27**		**46**		

Hospital Charges		$102,819 ± $87,766		$74,161 ± $36,177	0.054

Hospital Revenue		$65,953 ± $63,761		$45,795 ± $23,514	0.057

Total Hospital Costs		$40,910 ± $38,307		$25,200 ± $13,850	0.014

Direct Costs		$29,783 ± $27,977		$17,716 ± $9,854	0.009

Indirect Costs		$11,127 ± $10,346		$7,484 ± $4,093	0.037

Contribution Margin		$36,170 ± $41,985		$28,079 ± $16,770	0.249

Net Incomes		$25,043 ± $35,906		$20,595 ± $14,920	0.461

**Medicare/Medicaid**	**23**		**32**		

Hospital Charges		$100,738 ± $100,621		$81,605 ± $48,855	0.354

Hospital Revenue		$31,183 ± $30,749		$21,765 ± $13,196	0.127

Total Hospital Costs		$38,876 ± $37,209		$29,047 ± $17,719	0.197

Direct Costs		$28,147 ± $27,681		$20,765 ± $14,180	0.201

Indirect Costs		$10,729 ± $9,597		$8,282 ± $3,933	0.199

Contribution Margin		$3,035 ± $9,950		$1,000 ± $7,492	0.391

Net Income		$-7,693 ± $13,309		$-7,281 ± $9,277	0.893

## Discussion

Emergency department physician activation of the catheterization lab and immediate transfer of the patient to an immediately available catheterization lab by an in-house nursing team leads to a substantial reduction in door-to-balloon time and reductions in myocardial infarct size and decreased length of stay [14]. With this improvement in quality, total hospital costs decreased substantially for the initial hospitalization and subsequent care within one-year post-hospital discharge. However, payers had a nearly $10,000 reduction in average payment per admission to our hospital with the reductions in total payments increasing to more than $14,000 per patient in the one-year follow-up. Thus, the decrease in total hospital costs (Table 1) was offset by a nearly identical reduction in insurance payments leading to no change in hospital net income. Under current reimbursement practice, improvements in quality by reducing door-to-balloon time lead to considerable financial benefits which go entirely to payers.

As our study extended over nearly two years, the impact of the introduction of the improved heart attack care model on referral patterns could be a confounder in our results. However, until June 2007, the hospital followed a strict internal and external embargo on communications and marketing related to the new heart attack program. Thus, there were no internal communications or external marketing of the heart attack program until after the publication of the original article in Circulation. The hospital did develop a marketing campaign to the community regarding the program but this was not introduced until January 2008. Thus, for the time period of the data of this study (October 2004 to June 2006), only the hospital clinical staff was aware of the existence of the program. Thus, we do not believe there is any confounding of our results from this issue, as the community remained unaware of the heart attack program's existence.

One explanation of our findings could be that more patients had "surgical" anatomy during the Cardiology Activation/Routine Transfer period leading to more patients assigned a surgery DRG, resulting in increased resource utilization. Thus, our results would simply be due from imbalances of baseline characteristics between the two time periods. However, this was not the case as the proportion of patients undergoing coronary artery bypass grafting for complete revascularization was similar between the two time periods. In addition, "surgery" DRG's include not only coronary-artery bypass grafting (DRG 106) but also ventricular assist device placement (DRG 103), valve replacement (DRG 104), and intra-aortic balloon pump placement (DRG 110) (Figure 2). Review of the surgery DRG's and of the highest resource utilization cases reveal that surgery (including non-CABG procedures) was commonly used in response to the development of post-myocardial infarction cardiogenic shock and not simply for coronary artery bypass grafting to allow for complete revascularization (Table 3). We have shown previously that our ED Activation/Immediate Transfer protocol leads to a substantial reduction in myocardial infarct size [14], and we believe that this may lead to a resulting reduction in the incidence and severity of cardiogenic shock.

From a hospital perspective, a "business case" for quality has been advocated with the assertion that improved quality will decrease the costs associated with delivering patient care and improve hospital profitability [6-9]. However, most studies have focused primarily on the effects of quality improvement on hospital costs with the assumption that reductions in cost would lead directly to improved profitability [16]. These analyses fail to account for the impact of quality improvement on hospital revenue, which also directly influences hospital profitability. Although the quality improvement of reducing door-to-balloon time did in fact lead to substantial reductions in total hospital costs per admission, our data show that it also dramatically decreased reimbursement levels completely negating what would otherwise be seen as quite large hospital cost reductions. Thus, quality improvement can have unintended consequences and paradoxically worsen hospital profitability. In fact, similar results were experienced by Intermountain Healthcare which found its profit margin on Medicare patients with pneumonia disappear with implementation of extensive quality improvement [17]. In addition, studies have focused on comparisons of profitability between patients with high quality and low quality care with the expectation that quality improvement can shift the financial performance of costly low quality care to that of high quality care [7,8]. However, as shown in Figure 4, reducing door-to-balloon time decreased revenue more than costs leading to a modest decrease in hospital net income. For these reasons, it is difficult to make a "business case" for quality improvement through reducing door-to-balloon time from a hospital perspective.

The Institute of Medicine has recommended that payment systems financially reward the delivery of high quality care [1]. However, current hospital reimbursement practices have limited ability to reward improvements in hospital quality [1,18]. Specifically, for reducing door-to-balloon time, current reimbursement practices financially penalize hospitals for quality improvement as shown in our study. Most hospitals set their charges in a manner directly proportional to their underlying costs [19,20]. Thus, a quality improvement such as reducing door-to-balloon time, which reduces the hospital's costs, will typically automatically reduce the hospital's charges. Although their importance in hospital payments has diminished, hospital charges remain a component of many private payer contracts and are explicitly used in formulas to calculate outlier payments for Medicare and Medicaid and certain private payers [20,21]. Thus, the reduction in hospital charges will automatically reduce payments from many private payers contracts. Furthermore, the reduction in hospital charges will cause a reduction in outlier payments as seen in our study (Figure 1) [21].

With the advent of the prospective payment system, fixed payments to hospitals were intended to encourage hospitals to improve efficiency and quality resulting in financial benefits to their bottom line [22]. However, as seen in Figure 2, a patient with STEMI can have a wide variation in assigned DRG depending on the in-hospital clinical course ultimately leading to a nearly 20-fold variation in Medicare payment. Our study reveals that an improvement in door-to-balloon time leads to a decrease in DRG relative weight. Since Medicare/Medicaid payments are directly linked to DRG relative weight, hospital payments from Medicare/Medicaid dropped by more than $7,000 per hospital admission, which was proportionally similar to the reduction seen with private payers. In addition, the prospective payment system was designed primarily to reflect the hospital's costs of taking care of patients [23]. Thus, since our costs decreased, the payment system, in accordance with its specific design, reduced our hospital's reimbursements. These findings underscore major structural limitations in the prospective payment system when it comes to rewarding quality in acute myocardial infarction care. With the advent in 2008 of Medicare Severity DRG's, Medicare's linkage between costs and payment will further increase [24]. In addition, as more private payers adopt DRG based fixed payments similar to Medicare, this structural limitation in payment for improved quality will become more pervasive [20].

Purchasers and payers of healthcare have asserted that improved quality can reduce the cost of care [5]. Our study validates the relationship between reducing door to balloon time and reducing costs from a payer perspective. Yet, our study highlights a shortcoming of current reimbursement practices where the financial investments in quality programs by providers lead to large financial benefits to other parties [25]. However, given the potential large financial benefits to payers, greater emphasis on improving door-to-balloon time in pay for performance may be warranted to increase the economic rate of return of these programs. To be successful, a mechanism for shifting the benefits from payers to hospitals will be necessary [25]. Specifically, reduction in door-to-balloon time may be an appropriate target for innovative programs such as funding of startup costs by payers [26]. It also may be an appropriate area for the development of gain-sharing programs between payers, hospitals, and physicians [27].

### Limitations

Our results reflect the experience of one tertiary care community hospital and may not be applicable to other types of institutions or the healthcare system as a whole. Further studies involving other institutions and government and private payer databases will be required for confirmation of our results. Hospitals with different payer mixes and hospitals outside the United States may have different results. Although the two cohorts were similar from a demographic, presentation, and treatment standpoint, hidden baseline differences cannot be completely accounted for between the two time periods due to the non-randomized nature of our study. Our study failed to account for startup and maintenance costs. Addition of these costs would further increase the financial losses to the hospital. Our study failed to account for potential secondary financial benefits to our hospital for reducing door-to-balloon time such as enhanced reputation, improved patient throughput due to decreased length of stay, or the possibility of improved future reimbursements due to improved quality of care. Finally, our study reflects financial outcomes from reducing door-to-balloon time with emergency department physician activation of the catheterization lab and an immediate transfer process; improvements in door-to-balloon time by other methods may lead to different results.

## Conclusion

Emergency department activation of the catheterization lab and immediate transfer of the patient to an immediately available catheterization lab by an in-house transfer team led to dramatic reductions in door-to-balloon time with resulting decreases in myocardial infarction size and length of stay. Irrespective of the results of this financial study, widespread adoption of this strategy by cardiologists and hospitals is warranted due to its clinical benefits to patients with STEMI. From a payer perspective, this specific improvement in quality does dramatically reduce the overall cost of care. However, from a hospital perspective, there is limited financial incentive to adopt this program. To provide financial incentive to adopt this quality improvement, payment mechanisms designed to financially support the adoption of this program and to shift the economic benefits from payers to hospitals are needed.

An electronic copy of the order set used in the EHART^® ^protocol is available for download at .

## Abbreviations

CMS: Centers for Medicare and Medicaid Services; STEMI: ST-elevation myocardial infarction; ED: Emergency Department; DRG: Diagnosis-Related Group; CABG: coronary artery bypass grafting

## Competing interests

MLJ, JBG, and HT are employees of St. Francis Hospital and Health Centers. UNK, MBK, SRS, and WJB are employees of Indiana Heart Physicians. RT serves as the medical director for the St. Francis Emergency Department; his group, Emergency Physicians of Indianapolis, receives compensation for his work in this position. RT has also served as vice president of the medical staff of St. Francis Hospital from 2007 to 2008 and received compensation for this position. WJB served as medical director of the St. Francis Heart Center from 2007 to 2008 and received compensation for this position.

## Authors' contributions

UNK conceived and designed the study. UNK, MLJ, JBG, and HT acquired the clinical and financial data. UNK and CR performed the statistical analysis. All authors were involved in the analysis and interpretation of data. UNK drafted the manuscript. All authors revised the manuscript for important intellectual content. All authors have given final approval of the manuscript.

## Pre-publication history

The pre-publication history for this paper can be accessed here:



## Supplementary Material

Additional file 1 **Data With Inclusion of Self-Pay Accounts**. A secondary analysis was performed with inclusion of self-pay patients. When all patients including self-pay were analyzed, the overall pattern of charges, revenues, and costs was similar to the primary analysis. Click here for file
