# Supplementary material for: Financial impact of reducing door-to-balloon time in ST-elevation myocardial infarction: a single hospital experience
Source: BMC Cardiovasc Disord. 2009 Jul 26;9:32. doi: 10.1186/1471-2261-9-32 (PMC2731056; doi:10.1186/1471-2261-9-32)
Supplement: Additional file 1 — Data With Inclusion of Self-Pay Accounts. A secondary analysis was performed with inclusion of self-pay patients. When all patients including self-pay were analyzed, the overall pattern of charges, revenues, and costs was similar to the primary analysis. [file 1471-2261-9-32-S1.doc]

**Hospital Revenues, Costs, and Profit Margins With Inclusion of Self-Pay Patients**

When all patients including self-pay were analyzed, the overall pattern of charges, revenues, and costs was similar to the primary analysis.

|  |  | Cardiology Activation  Routine Transfer  October 1, 2004-August 31, 2005  (N=60) |  | ED Activation  Immediate Transfer  September 1, 2005-June 26, 2006  (N=86) |  |
| --- | --- | --- | --- | --- | --- |
| **All Patients** | **60** |  | **86** |  |  |
| Hospital Charges |  | $68,002±$69,255 |  | $52,794±$22,795 | 0.06 |
| Hospital Revenue |  | $31,380±$35,771 |  | $23,562±$16,900 | 0.08 |
| Total Hospital Costs |  | $26,826±$29,497 |  | $17,804±$9,057 | 0.009 |
| Direct Costs |  | $19,586±$21,946 |  | $12,584±$6,665 | 0.006 |
| Cath Lab |  | $6,750±$3,550 |  | $5,373±$1,994 | 0.003 |
| Inpatient Nursing |  | $5,382±$8,726 |  | $3,603±$2,753 | 0.079 |
| Surgery |  | $2,833±$10,183 |  | $373±$1,808 | 0.03 |
| Pharmacy |  | $2,414±$2,670 |  | $1,786±$1,921 | 0.101 |
| Respiratory |  | $586±$1,705 |  | $261±$932 | 0.14 |
| Lab |  | $455±$741 |  | $193±$160 | 0.002 |
| Emergency Room |  | $423±$135 |  | $504±$148 | <0.001 |
| Cardiology |  | $226±$371 |  | $263±$157 | 0.4 |
| Imaging |  | $205±$364 |  | $87±$160 | 0.009 |
| Supplies |  | $202±$322 |  | $129±$203 | 0.097 |
| Other |  | $111±$300 |  | $11±$52 | 0.003 |
| Indirect Costs |  | $7,240±$7,571 |  | $5,220±$2,518 | 0.023 |
| Contribution Margin |  | $11,795±$20,174 |  | $10,977±$14,972 | 0.779 |
| Net Income |  | $4,554±18,006 |  | $5,758±$14,840 | 0.66 |

**Hospital Revenues, Costs, and Profit Margins for Self-Pay Patients**

The self-pay net income data is potentially misleading as there were two sizeable hospital payments in the Cardiology Activation/Routine Transfer period while there were none in the ED Activation/Immediate Transfer (see Hospital Revenue Above). Exclusion of these payments would show that net income from self-pay patients, instead of decreasing, increased from -$18,665 per admission to -$12,593 per admission signifying the benefit of improved hospital cost structure on uncompensated care.

|  |  | Cardiology Activation  Routine Transfer  October 1, 2004-August 31, 2005  (N=60) |  | ED Activation  Immediate Transfer  September 1, 2005-June 26, 2006  (N=86) |  |
| --- | --- | --- | --- | --- | --- |
| **Self Pay** | **8** |  | **6** |  |  |
| Hospital Charges |  | $52,226±$12,826 |  | $43,192±$8,878 | 0.166 |
| Hospital Revenue |  | $7,571±$15,511 |  | $0±$0 | 0.260 |
| Total Hospital Costs |  | $18,665±$6,152 |  | $12,593±$3,163 | 0.049 |
| Direct Costs |  | $13,429±$4,295 |  | $8,880±$2,728 | 0.043 |
| Indirect Costs |  | $5,236±$1,883 |  | $3,714±$951 | 0.097 |
| Contribution Margin |  | $-5,858±$17,571 |  | $-8,880±$2,728 | 0.687 |
| Net Income |  | $-11,095±$18,685 |  | $-12,593±$3,163 | 0.851 |
